# Supplementary material for: Identification of photoperiod-induced specific miRNAs in the adrenal glands of Sunite sheep (Ovis aries)
Source: Front Vet Sci. 2022 Jul 22;9:888207. doi: 10.3389/fvets.2022.888207 (PMC9354845; doi:10.3389/fvets.2022.888207)
Supplement: Supplementary file 1 [file Data_Sheet_1.ZIP › Supplementary file/miRDeep2 file.zip!/miRDeep2 file/Novel_Structure/LP42A1/Novel_305.pdf]

Provisional ID	:	NC_019460.2_8618
Score total	:	2.6
Score for star read(s)	:	-1.3
Score for read counts	:	0
Score for mfe	:	2.3
Score for randfold	:	1.6
Score for cons. seed	:	
Total read count	:	26
Mature read count	:	26
Loop read count	:	0
Star read count	:	0

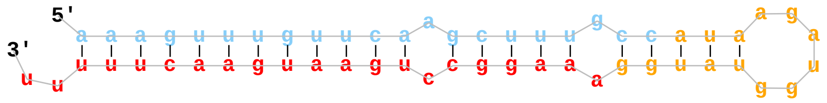

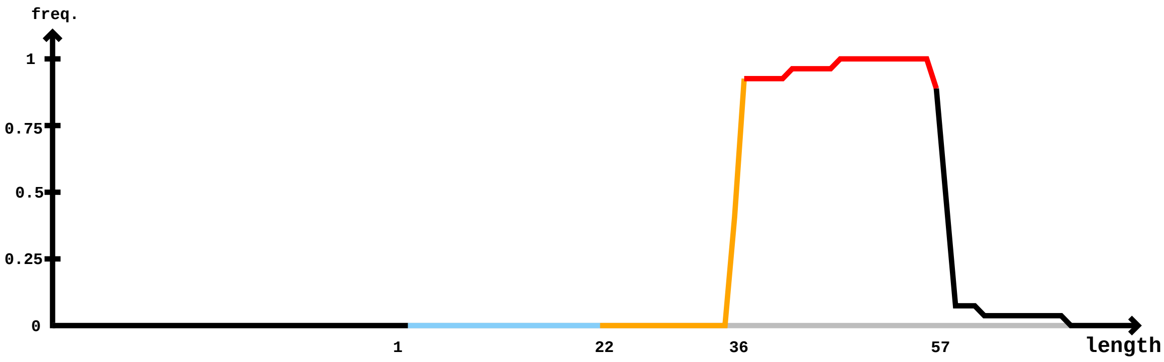

Star Mature

```
5'- gaacaacagcccuacuauuauauugguuggucaaaaaguuuguucaagcuuugccauaagaugguauggaaaggccugaaugaacuuuuugaccaacccaauauuucug   -3'    exp
    ...................(((((((((((((((((((((((((((.(((((.(((((......))))).))))).)))))))))))))))))))))))))))......    reads      mm         sample
    ....................................................................gaaaggccugaaugaacuuuuu...................    7          0          seq
    ....................................................................gaaaggccugaaugaacuuuuug..................    4          0          seq
    .....................................................................aaaggccugaaugaacuuuu....................    3          0          seq
    .....................................................................aaaggccugaaugaacuuuuu...................    7          0          seq
    .....................................................................aaaggccugaaugaacuuuuug..................    4          0          seq
    ..........................................................................ccugaaugaacuuuuugacU...............    1          1          seq
    ...............................................................................augaacuuuuugaccaacccaaGa......    1          1          seq
```
